# The Effects of Three and Six Sessions of Low Energy Extracorporeal Shockwave Therapy on Graft Incorporation and Knee Functions Post Anterior Cruciate Ligament Reconstruction

**DOI:** 10.5704/MOJ.2203.005

**Published:** 2022-03

**Authors:** M Rahim, FK Ooi, MT Shihabudin, CK Chen, AT Musa

**Affiliations:** 1 Exercise and Sports Science Programme, Universiti Sains Malaysia, Kubang Kerian, Malaysia; 2 School of Rehabilitation Sciences, Universiti Sultan Zainal Abidin, Terengganu, Malaysia; 3 Department of Orthopaedics, Universiti Sains Malaysia, Kubang Kerian, Malaysia; 4 Department of Radiology, Universiti Sains Malaysia, Kubang Kerian, Malaysia

**Keywords:** shockwave, graft incorporation, knee functions, anterior cruciate ligament reconstruction

## Abstract

**Introduction:**

One session of high energy extracorporeal shockwave therapy (ESWT) was found to improve the healing of anterior cruciate ligament (ACL) after reconstruction in animal and human studies. This study investigated the effects of three and six sessions of low energy ESWT on graft incorporation and knee functions post ACL reconstruction in humans.

**Materials and methods:**

Thirty participants with ACL injuries were recruited and assigned equally into three groups with 10 participants per group (n=10). Participants in the control group received physiotherapy alone without low energy ESWT. Participants in the 3ESWT group underwent three sessions of low energy ESWT (one session per week) combined with physiotherapy, and participants in the 6ESWT group received six sessions of low energy ESWT (one session per week) combined with physiotherapy. However, five participants were lost to follow-up. Evaluations of graft incorporation of the tibial tunnel using magnetic resonance (MRI) and Lysholm score were carried out before ACL reconstruction and after six months post ACL reconstruction.

**Results:**

The number of grafts with partial incorporation in the tibia tunnel in 6ESWT was significantly higher compared with the number of grafts with non-incorporation at six months post-operatively, X2 (1, N=9) =5.44, p =0.02. However, there was no significant difference between frequencies of graft incorporation in tibia tunnel in the control and 3ESWT groups, X2 (1, N=7) =3.57, p =0.06 and X2 (1, N=9) =2.78, p =0.10, respectively at 6 months postoperatively. Lysholm scores were significantly higher at 6 months post ACL reconstruction compared to the baseline value for each group (p<0.002, respectively). However, there was no significant difference in the Lysholm score between each group (F = 2.798, p = 0.083).

**Conclusions:**

Six sessions of low energy ESWT improved graft incorporation in the tibial tunnel. Both three and six sessions of low energy of ESWT does not affect the knee function score at six months post ACL reconstruction.

## Introduction

Anterior cruciate ligament (ACL) plays an important role to prevent anterior translation^1^, rotation of the knee^2^, and helps to stabilise the anterior aspect of the knee^1,3^. The mechanism of ACL injuries reported is due to the rotation of femur and tibia in opposite directions under full body weight. It happens when there is a sudden turning to change direction while running. Common symptoms of ACL injuries include pain, swelling, instability, and loss of function in severe cases^2^. As the consequence of ACL injury, athletes are adversely affected from training or competing^4^.

Conservative or surgical treatment of knee instability is indicated for regaining pre-injury function^5^. ACL reconstruction surgery aims to restore knee functions by achieving the tightest possible repair so that the reconstruction of the torn ligament can be the perfect biological replacement^6,7^. Anterior cruciate ligament (ACL) reconstruction is commonly performed by repositioning of a free tendon graft either from the patella or hamstring tendon into a bone tunnel^8^. The junction between the graft tissue is also known as ‘osteotendinous’ junction, ‘enthesis’, or ‘insertion site’^9^. In the long term, an ACL rupture can cause further intra-articular damage like meniscal tears, cartilage defects, and osteoarthritis. It has been reported that twothirds of primarily conservative treated patients decided to have ACL reconstruction after rehabilitation^10^.

A good ACL reconstruction outcome is said to have lesser knee laxity with a firm fixation^6^. Furthermore, the functional outcome of this surgery depends on the firm healing of graft incorporation with bone in the bone tunnel^8^. Some of the studies found that the graft incorporated well with the bone after implantation^11-14^ but other studies have shown contrasting results^15,16^. It is known that if the bone and tendon do not heal together, it may lead to future knee pathology. It has been estimated that 8-10% of ACL reconstructions result in recurrent instability and graft failure. Wang *et al*^8^ mentioned that two-third of the graft failed because of graft pull-out at 24 weeks post-surgery.

One of the factors that affect the surgical outcome of ACL reconstruction is the rate of graft healing^1^. In addition, it is claimed that the graft fixation using a hamstring tendon is less secure^3^. Different techniques and modalities are applied to accelerate the healing of new tendon implanted to the bone, however, inconsistent results have been reported^17-20^. Therefore, more studies that aim to reduce the risk of graft failure by increasing the rate of graft healing using external physical stimulus produced by the shockwave machine are warranted.

Several studies have demonstrated the effectiveness of extracorporeal shockwave therapy to treat various chronic musculoskeletal pathologies^21-27^. Sports injuries related to tendon-bone junction became an interest among the researchers due to its delay in healing for conservative treatment and increasing failure rate in surgical procedures. The complication of tendon and bone to blend is attributed to the non-homogenous structure in both types of tissues^28^. Although after a surgical intervention, the normal healing process of tendons is slow due to its low vascularity and low cellular composition in nature^29^. Thus, few strategies have been developed including using shockwave therapy as external mechanical stimulations to improve the natural tendon-bone healing.

From the current literature, shockwaves therapy is one of the rehabilitation modalities that has the potential to be applied to a tendon-bone junction to accelerate the healing process^8,30-38^. The mechanical stimuli produced by the shockwaves can induce physiological responses at the cellular level^39-41^. Several studies have previously reported the effectiveness of shockwave therapy on bone structure^26,41-43^ and tendon structure alone^44^.

Besides, shockwave therapy is listed as one of the biological therapies for tendon injuries of the knee joint^45,46^. Previous studies reported that extracorporeal shockwave therapy (ESWT) is beneficial to induce the ingrowth of neovascularization and improvement of blood supply at the bone-tendon junction^30,31^, promote tissue repair^8^, upregulation of angiogenic and osteogenic growth factors^47^ and increase cortical bone formation in acute fracture^48^. Additionally, it was also reported that ESWT is beneficial in promoting tendon-bone healing in the bone tunnel after ACL reconstruction in an animal^8^ and human study^32^. Nevertheless, a limited number of studies investigate the effects of ESWT on graft incorporation after ACL reconstruction.

The use of the Lysholm score as evaluation tool of knee functions has been reported to be more sensitive for the evaluation of activities of daily living and recreational or competitive sports^3^. High Lysholm score and International Knee Documentation Committee (IKDC) score are among the factors that influence the recreational sports player’s return to sports at the same level^49^. To date, based on a systematic review finding^46^, only Wang *et al*^32^ reported the effectiveness of ESWT post ACL reconstruction on physical function. Lysholm score was found to be better in participants who received ESWT compared with the participants who did not receive ESWT after one and two years of follow-up post hamstring autograft ACL reconstruction. However, in the similar study, the authors reported that the IKDC score was not affected by the intervention prescribed^32^.

Based on a systematic review study, there is limited evidence regarding the effects of ESWT following arthroscopic knee surgery^46^. Thus, additional studies are warranted to validate the previous study. This current study aimed to investigate the effects of three and six sessions of low energy ESWT on graft incorporation and knee functions in individual post ACL reconstruction.

## Materials and Methods

In this study, thirty participants with a single autograft hamstring reconstruction were recruited. However, twentyfive participants with a mean age of 26.8 ± 5.8 years old completed the present study. The participants were patients with an ACL tear, underwent primary single autograft hamstring ACL reconstruction, and the injury was caused mainly by involvement in sports activities. However, a few injuries were caused by other factors such as motor vehicle accidents and falls. Participants with primary single autograft patella tendon ACL reconstruction, revision ACL surgery, previous knee surgery which interfere with the knee functions, having multiple knee ligament injuries, medical problems, and participants on NSAIDs medication and/or consume calcium supplementation were excluded from this study.

Ethical approval to conduct this study was obtained from the Human Research Ethics Committee of Universiti Sains Malaysia (JEPeM) (USM/JEPeM/16090303) and Medical Research and Ethics Committee (MREC) (NMRR-18-95341870 (IIR)). This study was also registered with the National Medical Research Register (NMRR). All the procedures followed were in accordance with the ethical standards of the responsible committee on human experimentation (institutional or regional) and with the Helsinki Declaration of 1975, as revised in 1983. This is a quasi-experimental study and uses an opportunistic sampling method. A day before the operation, all potential participants were fully informed by the researcher about the nature of the experiments, purposes of the study, procedures, termination or withdrawal, potential risks, and benefits of participating in this study. The participation of the participants in this study is voluntarily, and they were allowed to withdraw from this study at any time during the study. If they agree to participate, they were asked to fill in the participant information and consent form. After that, participants were screened to determine the inclusion and exclusion criteria are met. The qualified participants then proceed to the next phase of this study, which was a grouping into control, 3ESWT, and 6ESWT groups.

Thirty male participants (N=30), with age ranging from 20 to 36 years old were recruited from the orthopaedic clinic of Hospital Universiti Sains Malaysia (HUSM) and Hospital Raja Perempuan Zainab II (HRPZ II) in Kelantan state of Malaysia. They were age matched and assigned into three groups using non-random method, i.e. natural experiment type of quasi-experimental design with 10 participants per group (n=10 per group). The participants have chosen to follow the normal protocol after ACL reconstruction (control group) or decided to try out the new therapy, i.e. shockwave therapy plus the normal protocol after ACL reconstruction (intervention groups) to suit with their convenience. Their preferable group was chosen based on their limitations such as transportation, location of their house and its distance from hospital, and commitment to attend all the scheduled follow-up sessions. The three groups were; i. Physiotherapy alone without ESWT control group (Control group), ii. Three sessions of low energy ESWT with one session per week combined with physiotherapy group (3ESWT group) and iii. Six sessions of the intervention of low energy ESWT with one session per week combined with physiotherapy group (6ESWT group).

All ACL reconstruction procedures were carried out by an orthopaedic surgeon in HUSM and HRPZ II based on a standard procedure. Both surgeons are accredited to perform these procedures. Intra-operatively, all participants underwent examination under anaesthesia to confirm the diagnosis of ACL tears. The reconstruction surgeries were carried out arthroscopically. Semitendinosus and gracilis tendon grafts were harvested and whip stitch were sutured at both ends. The grafts were folded in half and tensioned for 20 minutes. The tibial and femoral tunnels were drilled via the standard technique. The grafts were secured to the femoral end with a suspensory cortical button and the tibial end were secured with a bioabsorbable screw^2^.

The application of ESWT was administered to the participants once a week for three weeks (within week 7, week 8, and week 9 post ACL reconstruction) in the 3ESWT group. Meanwhile, for the 6ESWT group, the application of ESWT was administered to the participants once a week for six weeks (within week 7 until week 12 post ACL reconstruction). The ESWT was generated from a shockwave therapy device [ShockMaster 300, GymnaUniphy, Germany]. The treatment area was localised by palpation. The applicator of the ESWT was put directly on the skin region, perpendicular to the femoral tunnel at the lateral side of the knee joint, directed latero-medially, after applying the ultrasound gel. Then, shockwaves were released as the applicator button was pressed. The therapy was performed without anaesthesia. The total energy used was adapted from a study done by Wang, Huang and Pai^31^ i.e. 0.18 mJ/mm^2^ = 14kV for the 6ESWT group, similar total energy used by Wang *et al*^30^, Wang *et al*^8^, and Pavone *et al*^50^. While, half of the dosage, i.e. 0.09 mJ/mm^2^ was used for the 3ESWT group. It was stated in Rompe *et al*^51^ and Speed^52^ that ESWT is considered low energy with energy flux density (EFD) ranging from 0.08 – 0.27 mJ/mm^2^, medium energy with EFD ranged from 0.28 – 0.59 mJ/mm^2^, and high energy with EFD more than 0.60 mJ/mm^2^. ESWT was administered at 500 shocks, once per week for either 3 or 6 weeks. The frequency of treatment was selected based on previous studies^53-56^. After the intervention, the treatment area was inspected again to observe if any redness or hematoma occurred. Participants in the control group did not receive ESWT after ACL reconstruction.

This study aimed to deliver low energy of shockwave without anaesthesia but within pain tolerance for a few sessions of interventions. Based on the recommendation from the manufacturer of shockwave machine [ShockMaster 300, GymnaUniphy] and previous practice reported in the literature, one session of shockwave therapy per week for three and six weeks was scheduled. We decided to use the minimal tolerable intensity for our participants which was 1.5 bar^57,58^, since this was in the post-operative stage, the structures were mainly bone in nature, and the pain tolerance was low. The total number of shocks i.e. 500 shocks was similar to a previous animal study investigating the effect of the shockwave on bone healing around metal implants^8^.

The normal physiotherapy rehabilitation was performed by all the participants in all groups including continued wearing of knee braces and crutches post-operatively. All the participants were required to follow a physiotherapy regime prescribed by medical doctors and physiotherapists based on a standard ACL reconstruction rehabilitation protocol. The rehabilitation protocol was standardised by using an ‘accelerated rehabilitation program’ adapted from Shelbourne and Nitz^59^ as a guideline. There might be a slight modification of the standard protocol based on the capability and condition of the patients. Thus, the standard ACL reconstruction rehabilitation protocol used in this current study was the ‘modified accelerated rehabilitation program’ based on a protocol by Shelbourne and Nitz^59^.

Graft incorporation was determined by the quality of the bone-tendon interface and the signal quality of the ligament itself which can be observed on the image of Magnetic Resonance Imaging (MRI) at six months post ACL reconstruction. The result obtained from this evaluation determined if the treatment could accelerate the healing process of the new graft to bone six months post-operatively. The participants were screened after completing the screening forms which contained a few questions related to health status to ensure that they were safe and eligible to undergo MRI screening. The device used for this imaging was the Philips MRI machine [Achieva 3.0T TX, Netherlands]. The participants were asked to lie down on the MRI table. The affected knee was slightly bend at around 15° as the knee coil was placed at the joint. The total duration of this scan was around 40 to 45 minutes each session. The participants were given the MRI radiographer alert button during the scanning session. MRI image evaluation was performed by a trained and experienced musculoskeletal radiologist. He was blinded from the hypothesis of the study, participant’s group, and participant’s knee joint functional score. The images were coded and information that can identify each participant was removed. Only the research staffs of this study were able to link the code with each participant. The graft incorporation within the tunnels was characterised as ‘complete’, ‘incomplete’, or ‘none’. For complete incorporation, the bone-tendon interface was mature. For incomplete incorporation, the bone-tendon interface was partly mature and partly immature, while, for no incorporation, no evidence of a mature bone-tendon interface was found^60^. In a clinical setting, MRI was considered to be the best method in the evaluation of the healing of tendon to bone at the tendon-bone interface after ACL reconstruction in human subjects^32,61^. To determine the participants’ knee functions, Lysholm score were collected at pre-operatively (T0) and six months post ACL reconstruction (T4). Participants were asked to respond to the questions. The total scores would indicate the outcome of the ACL reconstruction, and they could be classified as excellent (95100), good (84-94), fair (65-83), and poor (≤64)^62^.

Statistical Package for Social Sciences (SPSS) version 26.0 was used for the statistical analysis. All the data are presented as mean ± standard deviation (SD). Mixed ANOVA was performed to determine the significant differences within and between groups. A ‘p’ value of <0.05 was considered statistically significant and was used for all the comparisons. If the mixed ANOVA test showed a significant difference, the post hoc test using the Bonferroni correction test was used to analyse the differences between each measurement. If the Bonferroni correction test showed a significant difference, mixed ANOVA test with the selected cases was used to analyse the differences between each measurement within each group. One way ANOVA with contrast test was used to determine the differences between the control group and the intervention groups, and the differences between both intervention groups. A Chi-square test for differences was performed to determine the significant differences for categorical data.

## Results

The participants’ demographic characteristics are presented in Table I. From a total of 30 participants recruited, 25 with a single autograft hamstring reconstruction and a mean age of 26.8 ± 5.8 years old completed the present study. Three participants from the control group and one participant from three sessions of shockwave therapy (3ESWT) and six sessions of shockwave therapy (6ESWT), respectively were excluded due to incomplete follow-up or had minor complications after surgery. The right leg was the most frequent leg prone to get injuries (60%). Most of the anterior cruciate ligament (ACL) torn was due to participation in football (60%) followed by futsal (20%), other factors (12%), takraw (4%), and volleyball (4%). The other mechanisms of injuries recorded were falling from a certain height and motor vehicle accidents (MVA).

**Table I:** Participants’ demographic characteristics

		Control group	3ESWT group	6ESWT group	N=25
		(n=7)	(n=9)	(n=9)	
Age (years) [Mean (SD)] (Range) Affected leg [Frequency (percentage)] Mechanism of injury		29.29 (4.39) / (22-36)	23.44 (5.05) / (20-36)	28.11 (6.39) / (21-36)	29.75 (5.80) / (20-36)
	Right knee	3	7	5	15 (60%)
	Left knee	4	2	4	10 (40%)
	Football	4	5	6	15 (60%)
	Futsal Takraw		3	2	5 (20%)
	Takraw		1		1 (4%)
	Volleyball			1	1 (4%)
	Others	3			3 (12%)

Abbreviations - Control: control group, 3ESWT: three sessions of extracorporeal shockwave therapy group, 6ESWT: six sessions of extracorporeal shockwave therapy group

**Fig. 1:**
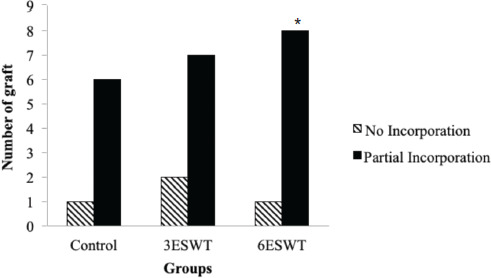
Frequencies of graft incorporation in the tibial tunnel in control, 3ESWT and 6ESWT groups at six months post-operatively.

**Fig. 2:**
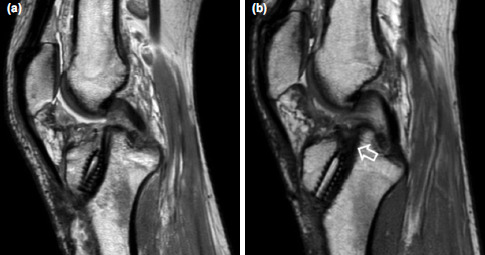
MRI images of tibial tunnel with graft in situ of a participant. (a) Pre-ESWT with no incorporation and, (b) was the post-ESWT that demonstrates partial incorporation (open arrow).

The result showed that the number of graft with partial incorporation in the tibia tunnel in the 6ESWT group was significantly higher compared with the number of the graft with non-incorporation at 6 months post-operatively, X2 (1, N=9) =5.44, p =0.02 (Fig 1). However, there was no significant difference between frequencies of graft incorporation in the tibia tunnel in control and 3ESWT group, X2 (1, N=7) =3.57, p =0.06 and X2 (1, N=9) =2.78, p=0.10, respectively at 6 months post-operatively. MRI images of tibial tunnel with graft in situ of a participant are illustrated in Fig. 2.

Control: control group

3ESWT: three sessions of extracorporeal shockwave therapy group

6ESWT: six sessions of extracorporeal shockwave therapy group

*, p<0.05 significantly different from ‘no incorporation’

**Fig. 3:**
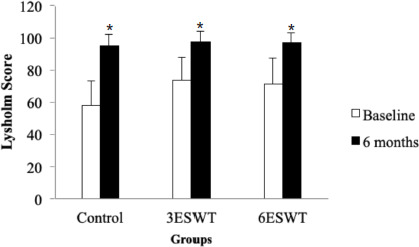
Means Lysholm score which reflect participant’s knee functions at baseline and six months post-operatively in control, 3ESWT and 6ESWT groups

Regarding Lysholm score, mixed ANOVA revealed that there was no significant interaction between time and group, F (2, 22) = 1.49, *p* = 0.247. In addition, there was no significant main effect of group observed, F (2, 22) = 2.798, *p* = 0.083. However, there was a significant main effect of time in Lysholm score, F (1, 22) = 74.41, *p*<0.001. Post hoc test using the Bonferroni correction revealed that there were significant differences in a comparison of time for all three groups; control [mean difference = -37.143, 95% CI (53.828, -20.458)], 3ESWT [mean difference = -23.556, 95% CI (-35.471, -11.641)], and 6ESWT [mean difference = 25.556, 95% CI (-38.157, -12.954)], p=0.002, respectively (Fig 3). The Lysholm scores were significantly higher at six months post-operatively compared with the Lysholm scores at baseline in all the groups. The present study also found that there were no complications such as redness, pain, and hematoma related to the ESWT interventions during and after the treatment.

Control: control group 3ESWT: three sessions of extracorporeal shockwave therapy group

6ESWT: six sessions of extracorporeal shockwave therapy group

*, p<0.05, significantly different from baseline value

## Discussion

To date, most human studies have reported the clinical effects of ESWT, meanwhile most animal studies reported the cellular effects of ESWT. ESWT has been speculated to accelerate the healing of transplanted tendon to bone via a few mechanisms. In animal studies, it was found that shockwave promotes osteogenesis^33-35^, increases regeneration of the fibrocartilage zone^33,34,36^, enhances bone remodelling in the delayed tendon-bone junction^33^, induces bone formation and healing of segmental femur defects associated with bone morphogenetic protein expression in callus^63^, enhances bone mass and bone strength after fracture^64^, induces neovascularization and improves blood supply at the tendonbone junction^30,31^, increases the number of trabecular bone around the tendons, and improves the contact between bone and tendon^8^, enhances the tensile strength of the tendon-bone interface^8,33,35^ and improves bone mineral status^33^.

The effects of low energy of ESWT on tendon-bone junction in animal was studied by Wang *et al*^8^ who applied one session of low energy of ESWT on tendon-bone junction after ACL reconstruction surgery in rabbits. They found that there was significantly more trabecular bone around the tendon, better incorporation of the tendon to bone, and higher tensile strength in the intervention group compared to the control group. Nevertheless, similar modes of graft failure were noted between the two groups. In other animal studies, high energy shockwave therapy has been reported to promote new bone formation^33,34^, higher expressions of VEGF^34^ and regeneration of the fibrocartilage zone^33,34,36^ in the delayed tendon-bone junction healing after partial patellectomy compared to the control group. Qin *et al*^34^ found that scar tissues were formed at the tendon-bone junction healing site in the control group after eight weeks postoperatively. Additionally, Wang *et al*^33^ found that there was significantly higher bone mineral status, tensile load and strength in the intervention group compared to the control group.

A comparison study by Chow *et al*^35^ showed that low energy shockwave therapy was as effective as high energy shockwave therapy in the treatment of delayed tendon-bone junction healing after partial patellectomy. They found that both intervention groups have larger bone areas, greater new bone volume, and significantly higher failure load at the tendon-bone healing junction than the control group at week 12 post-intervention. The results of these abovementioned studies were based on small animal i.e. rats/rabbits/dogs. The anatomy and physiology of the knee in those animals may not necessarily resemble the human subject. Therefore, the effects of ESWT in human subjects may be different from animals.

Several previous studies have provided evidence that few sessions of shockwave therapy might accelerate the enthesis healing by reducing the pain level, improved functional activities, including the participants’ quality of life^57,65-67^. Based on systematic reviews^45,46^, only one study used Magnetic Resonance Imaging (MRI) to investigate the effect of ESWT on tibial tunnel enlargement diameter after ACL reconstruction in humans^32^. In their study, high energy shockwave was applied once immediately after the operation with the same anaesthesia by focus type shockwave. Both diagnostic methods, i.e. radiograph and MRI presented a smaller tibia tunnel after two years and significantly decreased in tibia tunnel enlargement after six months and two consecutive years post-operatively. Based on the outcome, ESWT has the potential to promote tendon-bone junction healing at two years post ACL reconstruction surgery.

However, to date, studies using radio-imaging such as MRI to show the progress of graft incorporation healing especially after ACL reconstruction in humans are still lacking. Therefore, the present study was carried out. The main finding of the current study was that the number of the graft with partial incorporation in the tibia tunnel was significantly higher compared with the number of the graft with nonincorporation at six months post-operatively in the 6ESWT group. The current study also found that the number of the graft with partial incorporation in the tibia tunnel was higher compared with the number of the graft with nonincorporation at six months post-operatively in the control and 3ESWT group, respectively, however, it was not significant statistically. These findings showed that six sessions of low energy of ESWT may be effective to accelerate the graft healing by inducing graft incorporation post ACL reconstruction in humans. These findings also showed that graft incorporation of each participant responded differently towards the ESWT intervention and the number of ESWT sessions. Thus, it was speculated that the mechanism of ESWT on graft incorporation in humans may be similar with the mechanism in the animal studies.

This present study also found that knee function scores were significantly improved in the control, 3ESWT, and 6ESWT groups at 6 months post-operatively compared with the knee function scores at baseline, p=0.002, respectively. However, there was no significant difference in the Lysholm score between each group (F = 2.798, p = 0.083). Similar findings were observed in a previous study by Mohamed and Gaddur^68^, which was carried out to compare the effects of two different dosages of radial shockwave therapy (rESWT) on Jumper’s knee. The participants in the intervention group received 1000 shocks of rESWT with 2.0 bar intensity, and 20Hz frequency and were applied twice a week for 4 weeks. Meanwhile, the participants in the control group received 2000 shocks of rESWT with 2.0 bar intensity and 20Hz frequency were applied once a week for 4 weeks by using the same device. That study showed that physical functions were improved in both control and intervention groups but not significantly different. However, the increment of physical function score was significantly notable in the experimental group at 4th week compared to 3rd week post-intervention.

Wheeler^69^ reported that a combination of three sessions of ESWT with rehabilitation in patients with chronic plantar fasciitis was effective in improving the local function of foot and ankle but not the global function after six weeks, three months, and six months post shockwave therapy compared with baseline value. The energy dose was varied and specific to the individual, and different between sessions as the researchers applied ‘maximal comfortably tolerated’ energy dose for each participant.

Contradict with the findings from the present study, some researchers found that ESWT contributes to the improvement of the physical function of the participants. The physical ability of participants with calcific tendonitis of the shoulder, chronic Achilles tendinopathy, and lateral epicondylitis was improved compared to baseline value at one-year post ESWT^70^. In another study, it has been found that the effects of ESWT on functional outcomes among participants with knee osteoarthritis were dose-dependent. Both low and medium energy ESWT improved knee function compared with the baseline value. However, at week 1, 4, and 12, greater improvement was found in the medium energy group compared with the low energy group^71^. Nevertheless, there was no control group enrolled in these previous studies.

A few comparative studies have been done to investigate the effectiveness of ESWT on physical function among participants with knee osteoarthritis. In a retrospective study, radial ESWT was effective in relieving symptoms of knee OA, including improving knee functions which was evaluated using total WOMAC score. After 5 sessions of ESWT with 3 days interval, ESWT showed greater beneficial effects compared with laser therapy at week 6 and week 12 post-therapy^72^. In a comparison study, 5 sessions of shockwave therapy resulted in an improvement in physical function in the WOMAC sub-score among participants with knee osteoarthritis at week 5 post-intervention compared to the kinesiotherapy group. The shockwave therapy session was carried out once a week for five weeks with one-week interval^73^. In a randomised control trial study, it was found that shockwave therapy was more effective than ultrasound therapy in improving the physical function among participants with osteoarthritis knee. The ESWT group received 1000 pulses during the first treatment, 1500 during the second and the third treatments, and 2000 during the fourth and the fifth treatments, respectively (pressure, 2.5 bar; frequency, 8Hz; energy density, 0.4 mJ/mm^2^). The participants attended five sessions of shockwave therapy, i.e. one session per week for five weeks with weekly intervals. The researchers used Knee injury and Osteoarthritis Outcome Score (KOOS) as a primary outcome measure to assess physical function among participants^74^. These studies showed that ESWT was superior to laser, kinesiotherapy, and ultrasound in improving physical function among participants with osteoarthritic knee.

A randomised controlled trial study found that four sessions of a low dose ESWT, i.e. once per week for four consecutive weeks significantly improved the physical function level of participants with the osteoarthritic knee at week 5 and week 12 post-shockwave therapy in the intervention group compared with the control group. The participants in the intervention group received a total of 2000 pulses of 8Hz frequency at 2.5 bars while the participants in the control group received sham shockwave therapy with a similar protocol of ESWT but using 0.2 bars of air pressure^75^.

A meta-analysis study concluded that the physical function of participants with osteoarthritis knee using Western Ontario and McMaster Universities Osteoarthritis Index (WOMAC) was significantly improved at 4 weeks, 8 weeks, and 12 weeks post low-intensity shockwave therapy compared to the control group^76^. In a long term follow-up study, three sessions of low energy radial ESWT improved the functional outcome of the participants with insertional and non-insertional Achilles tendinopathy after two years post-intervention. The researchers applied 2500 pulses per treatment and were administered at weekly intervals. The ESWT dosages were 1.5 bar with 10Hz for the first 500 pulses and were increased to 2.5 bar for the remaining of 2000 pulses^77^. Most of the previous studies reported the effects of ESWT on physical function among participants with chronic diseases such as jumper’s knee, chronic plantar fasciitis, osteoarthritic knee, calcific tendonitis of the shoulder, chronic Achilles tendinopathy, and lateral epicondylitis. The enhancement in physical function can be seen as early as one-week post-ESWT in these previous studies.

There is limited evidence on the effectiveness of ESWT on local function or global physical function among participants after surgical procedure. Wang *et al*^32^ showed that ESWT was effective in improving participants’ functional score at one and two years post-operatively. It was postulated that variation in conditions treated, different methods of ESWT application, dosage or energy used, number of ESWT sessions, type of shockwave machine, and duration of follow-up period may lead to inconsistent results. Besides, it was speculated that improvement in physical function following ESWT post-surgical procedure can only be observed after a longer follow-up period. The outcome of physical function may be influenced by patients’ selfmotivation and adherence to the rehabilitation sessions as the confounding factors.

## Conclusions

There are a few limitations in this current study. The participants in the present study were not randomly selected and allocated into groups as limited numbers of patients met the inclusion criteria and not many of them agreed to participate in this study. Besides, the commitment, transportation, and logistics problems are the factors that need to be considered when allocating them into control or the intervention groups. Thus, to fulfil the required number of patients in the study period, randomisation cannot be done. Due to the aforementioned problems, the sample size for this study was relatively small. Nevertheless, the sample size calculation has been performed based on a previous related study. In addition, the duration of follow-up was relatively short. Thus, future studies with long term followup and randomisation with large sample size is warranted to confirm and validate these findings.

As a conclusion, six sessions of low energy ESWT improved graft incorporation in the tibial tunnel. Both three and six sessions of low energy ESWT did not elicit additional benefits on knee function score at 6 months post ACL reconstruction.
